# The relationship between resting energy expenditure and thyroid hormones in response to short-term weight loss in severe obesity

**DOI:** 10.1371/journal.pone.0205293

**Published:** 2018-10-19

**Authors:** Paolo Marzullo, Alessandro Minocci, Chiara Mele, Rezene Fessehatsion, Mariantonella Tagliaferri, Loredana Pagano, Massimo Scacchi, Gianluca Aimaretti, Alessandro Sartorio

**Affiliations:** 1 IRCCS Istituto Auxologico Italiano, Division of General Medicine, Piancavallo, Verbania, Italy; 2 Università del Piemonte Orientale, Department of Translational Medicine, Novara, Italy; 3 IRCCS Istituto Auxologico Italiano, Division of Metabolic Diseases, Piancavallo, Verbania, Italy; 4 University of Turin, Department of Medical Sciences, Turin, Italy; 5 University of Milan, Department of Clinical Sciences and Community Health, Milan, Italy; 6 IRCCS Istituto Auxologico Italiano, Experimental Laboratory for Auxo-Endocrinological Research, Piancavallo, Verbania, Italy; Medical University Innsbruck, AUSTRIA

## Abstract

**Background:**

Regulating thermogenesis is a major task of thyroid hormones (THs), and involves TH-responsive energetic processes at the central and peripheral level. In severe obesity, little is known on the relationship between THs and resting energy expenditure (REE) before and after weight loss.

**Methods:**

We enrolled 100 euthyroid subjects with severe obesity who were equally distributed between genders. Each was examined before and after completion of a 4-wk inpatient multidisciplinary dieting program and subjected to measurement of thyroid function, REE, fat-free mass (FFM, kg) and percent fat mass (FM).

**Results:**

Baseline REE was lower than predicted in 70 obese patients, and overall associated with BMI, FFM and FM but not thyroid-related parameters. By the study end, both BMI and REE decreased (5.5% and 4.1%, p<0.001 vs. baseline) and their percent changes were significantly associated (p<0.05), while no association related percent changes of REE and FFM or FM. Individually, REE decreased in 66 and increased in 34 patients irrespective of gender, BMI and body composition. Weight loss significantly impacted TSH (-6.3%), FT3 (-3.3%) and FT4 levels (3.9%; p<0.001 for all). By the study end, a significant correlation became evident between REE and FT4 (r = 0.42, p<0.001) as well as FT3 (r = 0.24, p<0.05). In stepwise multivariable regression analysis, however, neither THs nor body composition entered the regression equation for REE response to weight loss.

**Conclusions:**

In severe obesity, short-term weight loss discloses a positive relationship between REE and THs.

## Introduction

The functions of the hypothalamic-pituitary-thyroid (HPT) axis are influenced by environmental and physiological factors, the most relevant of which are external temperature, iodine intake, reproduction and aging [1]. Increasing attention has recently focused on the bidirectional association linking the HPT axis to fat accumulation [2], which encompasses control of thermogenesis and body weight, lipid metabolism, thyroid hormone (TH) balance and thyroid morphology [3]. It is generally acknowledged that TSH levels increase with accumulating adiposity in euthyroid subjects spanning a wide range of BMIs [4]. Adiposity is also capable of influencing circulating THs, and several authors reported a shift toward low-normal FT4 and high-normal FT3 concentrations along with increasing BMI [5–9]. Typically, these adaptive fluctuations can be reversed by weight loss [10–14]. Although the mechanisms responsible for this changes are incompletely understood, the main neuroendocrine signal governing the response of the HPT axis to adiposity involves actions of leptin on TRH activity in the brain and hindbrain [10,15–18]

The effects of HTP on facultative thermogenesis entail both central and peripheral actions, on cellular processes governing triiodothyronine-responsive energetic mechanisms [19–23]. Excess deviations in energy expenditure often involve medical conditions affecting the HPT axis, such as hypo- and hyperthyroidism or cachexia [24]. It is recognized that, both in obese and lean subjects, caloric restriction potently blunts energy expenditure at rest as a consequence of first-phase losses in fat-free mass and late-phase losses in fat mass [24–27]. Adaptive thermogenesis, the inherent modification responsible for homeothermic variations of energy expenditure during and after weight loss, is statistically sustained by prediction models that are conventionally built on fat-free mass and fat mass [28]. Nonetheless, such equations cannot be entirely predicted by body composition-related parameters in people with varying degrees of adiposity, and more likely involve organ-tissue based models, which remain problematic to compute [29–30]. Recognizing that around 25% of REE is dependent on THs and that REE and THs are responsive to small changes of body weight within the euthyroid state [19,31,32], we sought to explore the effects of a short-term weight reducing program on the interplay between energy homeostasis and the HPT axis in subjects with severe obesity.

## Patients population

The study population was constituted by 100 consecutive euthyroid obese patients (BMI >30 kg/m^2^), classified as severely obese (50 females/50 males; mean age, 40.4±12.7 yr; mean BMI, 45.1±4.8 kg/m^2^, BMI range, 40–61 kg/m^2^). Subjects were recruited according to the inclusion criteria upon admission to our Institution for diagnostic workup and rehabilitation for severe obesity. The experimental procedure was approved by the ad hoc Ethical Research Committee of the Istituto Auxologico Italiano, Verbania, Italy, and written informed consent was obtained from the participants. The study protocol conformed to the guidelines of the European Convention on Human Rights and Biomedicine concerning biomedical research. Following baseline assessment, all participants underwent a 4-week inpatient study consisting of multidisciplinary weight loss program including the following: a) personalized diet, daily monitored by a dietician, formulated according to the Italian recommended daily allowances [33], entailing an energy intake corresponding to the 75% of the measured REE [34]; b) aerobic physical activity program, including two 30-min sessions/day of cycle ergometer pedaling, treadmill walking and stationery rowing, carried out for 5 days/week. The intensity of exercise was set at an average heart rate between 60% and 80% of the individual’s maximum heart rate; c) psychological and nutritional counselling. During the study period, patients were fed a balanced diet (30% lipids, 52% carbohydrates, and 18% proteins). No patient was undergoing pharmacological therapies at the time of the study, and body weight had been stable for at least three months prior to study entry. Each part of the study was conducted under skilled medical surveillance and nursing. To reduce the detection bias, none of the study participants suffered from thyroid disease nor had been previously treated with medications potentially interfering with thyroid function. Patients with known cardiac disorders, peripheral edematous congestion, EKG and/or cardiac symptoms were during baseline screening on hospital admission were excluded from the study [35]. Other exclusion criteria included endocrine obesity, autoimmune or chronic inflammatory disorders, type 1 diabetes mellitus (T1DM) and T2DM, chronic obstructive pulmonary disease, history of neoplasms or degenerative diseases, previous chronic steroid treatment, kidney disorders, and liver disease.

## Body measurements

Both at the beginning and at the end of the study, all testing procedures were performed between 0800-0930am in fasting conditions and after voiding. At baseline, all patients underwent metabolic profiling and thyroid ultrasonography (US). Hormonal assessment, anthropometry data, as well as indices of body composition and REE were determined at baseline and at the end of the 4-week study period according to the study protocol. All subjects underwent body measurements wearing light underwear, in fasting conditions after voiding. Weight and height were measured to the nearest 0.1 kg and 0.1 cm, respectively. BMI was expressed as body mass (kilograms)/height (meters)^2^. Obesity was defined for BMI ≥30 kg/m^2^. Waist circumference was measured midway between the lowest rib and the top of the iliac crest after gentle expiration. Anthropometric data were expressed as the mean of two measurements.

Respiratory quotient (RQ; VO2/VCO2) and REE (kcal/24 h) were determined in a thermo-regulated room (22–24° C) by computed open-circuit indirect calorimetry, measuring resting oxygen uptake and resting carbon dioxide production by a ventilated canopy (Sensormedics; Milan, Italy) at 1-min intervals for 30 min and expressed as a 24-h value. The test consists of making each patient lie down relaxed on a comfortable armchair, with the head under a transparent hood connected to a pump, which applies an adjustable ventilation through it. Exhaled gas dilutes with the fresh air ventilated under the hood and a sample of this mixture is conveyed to the analyzers, through a capillary tube and analyzed. Ambient and diluted fractions of O2 and CO2 are measured for a known ventilation rate, and O2 consumption (VO2) and CO2 production (VCO2) are determined. Energy expenditure was calculated according to the Weir equation [36]: EE = 5.68 VO2 + 1.59 VCO2–2.17 N_u_. As short-term urinary collections to assess total nitrogen excretion (N_u_) may not be representative of the protein oxidized during the measurement itself, they were not be obtained in this study, and assumed to be 13g/24h [37]. REE was assessed at study entry and after 4-week weight loss program. Predicted REE (pREE; kcal/day) was calculated by the Harris-Benedict formula and was employed to calculate the REE/pREE ratio as a proxy of thermogenic potential, set as normal at 100% [34].

Fat mass and free fat mass were assessed by bio-impedance analysis (BIA, 101/S Akern; Florence, Italy). The two vector components of impedance (i.e. resistance and reactance) were obtained by single measurements; before each testing session, the external calibration of the instrument was checked with a calibration circuit of known impedance value. The mean coefficient of variation was 1% for within-day and 3% for weekly intra-individual measurements in the steady-state condition in either site and 2% for inter-operator variability. Patients with fluid overload according to vectorial analysis were excluded to minimize errors in estimating FBM and FFM [34].

To further account for thyroid abnormalities potentially relating to thyroid function, thyroid morphology and echogenicity were studied by real-time US device equipped with a linear transducer operating at 7.5 MHz (MyLab Class C, Esaote Biomedica, Genova, Italy). Thyroid echogenicity was assessed in comparison with neck muscles after excluding the potentially reflecting echoes from isthmus. When present, thyroid nodules were subjected to fine needle biopsy if considered suspicious according to guidelines [38].

## Laboratory

Thyroid function was tested by analysis of FT4, FT3, TSH, anti-Tg antibodies (TgAb) and anti-TPO antibodies (TPOAb) levels. Undiluted serum samples were assayed using an automated electrochemiluminescence assay system (Cobas 6000; Roche Diagnostics GmbH, Mannheim, Germany). The principle of the method is a two-site, solid-phase chemiluminescent sandwich immunoassay. Normal values were as follows: FT3, 1.8–4.4 ng/L; FT4, 8.0–19.0 ng/L; TSH, 0.27–4.2 mIU/L; TPOAb, less than 35 μU/L; TgAb, less than 40 μU/L.

## Statistical analysis

Data were tested for normality of distribution by the Kolmogorov-Smirnov test and log-transformed when needed, to correct for skewness. Variables of interest were considered as absolute values at the beginning and end of the study, and calculated as percent variations over baseline values [Δ = ((T_1_-T_0_)/T_0_)*100]. For comparative analyses, paired T-test for intra-individual comparisons and ANOVA between subgroups were used. Correlations between parameters of interest were tested by bivariate regression analyses, partial correlation analysis, and ANCOVA by the general linear model to control for potential interactions. The Bonferroni correction was used for multiple testing analyses. Gender was coded as 0 (females) and 1 (males). The presence of thyroid nodules at US was coded as 0 (no nodule) and 1 (≥1 nodule). Based on results of bivariate analysis and ANCOVA, a stepwise multivariable regression analysis was performed to assess the role of anthopometric variables, and their changes, on REE and thyroid function tests after weight loss. Two-sided p<0.05 was considered as statistically significant.

## Results

Results obtained at baseline and at the end of study are summarized in S1 Table. Thyroid function was normal in all patients except for two cases with slightly elevated TSH levels, who tested negative for TPOAb and TgAb and showed normal thyroid echogenicity at US. Thyroid was hypo-echoic in 11 cases and ≥1 nodule was detected in 60% of cases. Of 8 patients with nodules who underwent biopsy, one harbored a medullary thyroid carcinoma.

Baseline anthropometric assessment by calorimetry revealed lower than predicted REE (i.e., REE/pREE <100%) in 69 obese patients, who showed no differences in study parameters vs. patients with a higher than predicted REE (S2 Table). In gender-stratified analysis (S3 Table), men were slightly older and showed higher adiposity, REE, and FT3 than women. In the population as a whole, REE was unrelated to parameters of thyroid function (S4 Table) and morphology while being, predictably, correlated with BMI (r = 0.31, p<0.01), FFM (r = 0.68, p<0.001), and percent FM (r = -0.25, p<0.05). Inverse correlations were seen between TSH and age (r = -0.25, p<0.05) and thyroid nodules (r = -0.33, p<0.001).

At the end of the 4-week study, mean BMI decreased by 5.5±1.8% and significant changes in thyroid function tests were documented (S1 Table). When the initial REE/pREE ratio was accounted for, weight loss was slightly weaker in subjects with a low vs. high REE/pREE (-5.3±1.6% vs -6.04±2.1%, p = 0.07). Alternatively, when the final REE/pREE results were accounted for, weight loss did not differ between patients with low vs high REE/pREE (-5.5±1.7% vs -5.7±2.2%, p = 0.8). By the end of treatment, absolute REE values decreased on average by 102±229 kcal/day, and similar percent changes in REE were observed between genders (S3 Table). Individually, percent REE responses to weight loss were variable as REE decreased in 66 patients by 0.14–29.8% and increased in the remainder by 0.25–35.6%. Comparative analysis between these subgroups (S5 Table) failed to reveal differences in thyroid function parameters, BMI or body composition, as well as gender prevalence, while obvious divergences in REE and its related variables were present. In considering patients with vs without biochemical and/or US signs of thyroid autoimmunity, a non-significant difference in delta REE was noted between subgroups (-2.4±8.6% vs. -4.5±11.3, p = 0.4). By the end of treatment, the association between REE and BMI (r = 0.24, p<0.05), FFM (r = 0.71, p<0.0001) and percent FM (r = -0.33, p<0.01) remained unaltered. A marginal but significant relationship was seen between ΔREE and ΔBMI (r = 0.22, p<0.05). Alternatively, there was no association between ΔREE or ΔREE/FFM and ΔFFM or ΔFM. Of note, weight loss disclosed a positive association between REE and FT4 (r = 0.42, p<0.001) as well as FT3 levels (r = 0.24, p<0.05) (S4 Table). Using the general linear model, which dummy codes categorical variables, we found no interaction between the association of REE with THs and the loss of FFM. Finally, ΔBMI was well correlated with ΔFT4 (r = -0.38, p<0.001), and border-line correlated with ΔTSH (r = 0.18, p = 0.07).

Further, to search for predictors of REE and THs response to weight loss, a stepwise multivariable regression was conducted using age, gender, baseline value of the variable of interest, baseline BMI, ΔBMI, ΔFFM and ΔFM as independent parameters. A number of different models were subsequently tested to avoid collinearity. ΔREE was only predicted by its baseline values (β = -0.59, p<0.001), while ΔTSH was best predicted by its baseline values (β = -0.30, p = 0.003) and ΔBMI (β = 0.20, p = 0.03); ΔFT4 was best predicted by its baseline values (β = -0.51, p<0.001), male gender (β = 0.36, p<0.001) and ΔBMI (β = -0.17, p<0.05); ΔFT3 was predicted by its baseline values (β = -0.58, p<0.001) and male gender (β = 0.33, p<0.001); finally, ΔFT3/FT4 was predicted by ΔBMI (β = 0.31, p<0.01).

## Discussion

Growing attention has recently focused on the ability of weight loss to restore thyroid function parameters in obesity. In the present study, we documented an association between THs and REE in severely obese euthyroid patients undergoing a short-term multidisciplinary weight loss program. While it could be gathered that weight loss disentangles the relationship between thyroid activity and REE, from the standpoint of energy balance and weight control our short-term results should be confirmed in long-term studies to add further evidence of an activating relationship between thyroid, adiposity and energy homeostasis during weight loss.

FFM exerts a predictive effect on REE and its inter-individual variability over a broad range of BMIs [39]. In obese subjects, values of absolute resting and total energy expenditure are conventionally higher than in lean controls, but these differences disappear when FFM is accounted for, suggesting that intrinsic energy expenditure is not altered in obese individuals [40]. On the other hand, qualitative and quantitative changes of FFM instigated by caloric restriction are capable of decreasing REE to values below the prediction models, implying the intervention of body composition-unrelated components [27,30,41–45]. In this euthyroid cohort of severely obese patients we documented at enrolment a lower than predicted REE in nearly 70% of patients, possibly reflective of a weak thermogenic potential [24]. Nonetheless, REE and FFM were tightly associated and both decreased after weight loss but the magnitude of their losses was not interrelated, while a marginal but significant association related percent variations in REE and BMI. BMI decreased by 5.5% and promoted a decline in REE that was equivalent to 102±229 kcal/day. This figure seems proportional to the loss of 244–301 kcal/day reported by Leibel et al. in subjects achieving ≥10–20% weight loss during a high-fat caloric restriction regimen [46]. In individual analysis, however, REE increased a third of patients after weight loss. In comparison to patients with decreased REE, these patients showed similar gender distribution and equivalent changes in weight and body composition, but were generally older and harbored lower baseline REE both as absolute values and normalized for pREE or FFM. The reason/s for this variability in REE response to weight loss is only partly understood and possibly depends on differences in metabolically active organs [27,29]. A number of univariate and multivariate regression analyses were conducted to identify the determinant/s for this outcome, but the only predictor of REE response to weight loss was its initial value. Bearing in mind that the effects of body and/or organ mass as well as sympathetic tone activity were not investigated, our short-term result could suggest that a high baseline REE predisposes to proportionally larger reduction in REE due to losses from organs with high metabolic activity [29,45]. In turn, this would explain the association linking percent REE to BMI changes, but not to FFM and/or FM changes. At odds with these findings, a 12.7±2.2 wk study in overweight/severely obese women fed a high-protein low-fat 800–1000 calorie diet showed that absolute and relative values of REE were higher in patients with high vs low REE response [29]. Whether differences in population sample, obesity degree, study design, diet regimen, macronutrients composition, and protein sparing may play a role in the discrepancies between these and our results remains unclear. In addition, our study protocol included fixed physical activity session as standard component of inpatient anti-obesity protocol, but we cannot exclude that differences in individual propensity and/or effort made under exercise could play a role. Further more specifically designed studies should clarify the effect of physical activity regimens on REE during weight loss.

In addition to body composition-related variables, adaptive thermogenesis is modulated by metabolic, neuroendocrine, autonomic, and behavioral responses [47]. Changes in insulin [29], leptin [48], and sympathetic tone [49] all play a role on REE modifications after weight loss. Thyroid elicits its effects on energy expenditure by acting on white and brown adipocytes, spontaneous motor activity, mitochondria thermogenesis and hypothalamic control of the sympathetic nervous output to the brown adipose tissue [50,51]. In untreated obesity, the relationship between REE and thyroid function is generally null [52,53], and our baseline results confirm this gap. Adaptive thermogenesis in response to weight loss has been associated with changes in serum TSH or T3 in some but not all studies [29,45,48,49,54–56]. The current analysis revealed that weight loss, while producing diverging effects on TSH and FT3 on one side and FT4 on the other [3], disclosed a significant association between REE and FT4 as well as FT3, regardless of changes in body composition. This interaction suggests the intervention of body composition-independent neuroendocrine signals controlling energy metabolism via THs. Because our study did not include the measurement of circulating leptin, which is rapidly responsive to weight loss [57] and regulates the thermogenic activity through central mechanisms involving TRH [3,58,59] our results provide no insight on neuroendocrine control of TH-REE association during weight loss.

As weight loss significantly reduced TSH and FT3 levels, while enhancing FT4 ones, a lowering of type II deiodinase activity could be involved in a such variations [60]. It is known that the FT3/FT4 ratio reflects conversion of fT4 into fT3 through type 2 deiodinase activity [61], and this ratio increases in obesity likely due to increased deiodinase activity as a “compensatory mechanism” against weight accrual. In cross-sectional [62] and cross-sectional plus longitudinal studies [63] an association between the FT3/FT4 ratio and adiposity measures and insulin resistance was found, while FT3/FT4 decreased after bariatric surgery, suggesting a reduction in deiodinase II activity in the circumstance of weight loss. While our study confirms the reduction of the FT3/FT4 ratio after weight loss, it relationship with changes in REE was not significant, suggesting that deiodinase activity may not be directly involved in the relationship between thyroid function tests and REE after weight loss. However, changes in FT3 and FT4 could also be due to variations in serum levels of total T3 and T4, or thyroid hormones binding proteins, which were not measured in this study. Other study limitations should be acknowledged as potentially affecting our results. First, body composition was calculated from BIA. Although patients with fluid overload, which overestimates fat mass, were excluded from the study, BIA is indeed of modest diagnostic value when compared to more refined techniques, such as CT or MRI, and shows its limits mostly in abdominally obese subjects [64]. however, previous studies from our laboratory [65] and others [66] suggested that BIA results are more similar to DEXA and BOD POD results in severe obesity than in lean or overweight subjects. Secondly, our study duration was calculated conceivably to circumvent the period of 6–8 weeks required for the physiological resetting of the HPT axis [67,68]. Thus, the significance of our findings remains to be proved in longer studies. Thirdly, the standard multidisciplinary approach used herein comprised 5 weekly sessions of (non-vigorous) physical activity, which could mitigate the natural loss in FFM occurring with caloric restriction. However, vigorous exercise does not prevent the loss in FFM occurring during caloric restriction [45], suggesting that metabolic adaptation persists even upon combination treatments of obesity. Lastly, our study participants were selected as euthyroid and severely obese, such that current findings may not fully apply to people with normal bodyweight or mild obesity, as well as those with thyroid dysfunctions. Nevertheless, we consider the homogeneous study sample, the controlled inpatient regimen, the balanced diet and the controlled weight management schedule as potential points of strength of this study.

In conclusion, we observed an association between REE and thyroid hormones in severe obesity after a short-term, mildly hypocaloric multidisciplinary weight loss program. How thyroid hormone impacts energy expenditure during long-term calorie restriction warrants further investigation, as it could frustrate weight loss attempts of obese individuals. Long-term studies are awaited to add further evidence of an activating relationship between thyroid, adiposity and energy homeostasis during weight loss.

## Supporting information

S1 Table Baseline data in the obese population obtained at baseline and at the end of the 4-week study, and expressed as percent variation over baseline values. S1 Table legend. Significance between the two time points was obtained by paired T test and is depicted as: a, p<0.05; b, p<0.01; c, p<0.001. For abbreviations: BMI, body mass index; REE, resting energy expenditure; pREE, predicted REE; FM, fat mass; FFM, fat-free mass.
(DOCX)

S2 Table Data summary in the obese population at study entry stratified according to REE measured as lower vs higher than predicted REE. S2 Table legend: Significance between the two subgroups was obtained by ANOVA. For abbreviations: BMI, body mass index; REE, resting energy expenditure; pREE, predicted REE; FM, fat mass; FFM, fat-free mass.
(DOCX)

S3 Table Data summary obtained at baseline and at the end of the 4-week study in the obese population subgrouped by gender. S3 Table legend: Significance between genders at baseline and at the study-end time-points was assessed by ANOVA and is depicted as: a, p<0.05; b, p<0.01; c, p<0.001. Significance within genders between baseline and study-end time-points was assessed by paired T test and is depicted as: d, p<0.05; e, p<0.01; f, p<0.001. For abbreviations: BMI, body mass index; REE, resting energy expenditure; pREE, predicted REE; FM, fat mass; FFM, fat-free mass.
(DOCX)

S4 Table Bivariate correlation analysis between thyroid function parameters and REE at baseline and at the study end. S4 Table legend: For abbreviations: REE, resting energy expenditure.
(DOCX)

S5 Table Data summary in the obese population stratified according to percent REE variation below (decreased REE) or above (increased REE) baseline values recorded at the end of the study. S5 Table legend: Between-subgroup significance at each time point was assessed by ANOVA and expressed as: a, p<0.05; b, p<0.01; c, p<0.001. Within-subgroup significance between the two time points was assessed by paired T test and expressed as: d, p<0.05; e, p<0.01; f, p<0.001. For abbreviations: BMI, body mass index; REE, resting energy expenditure; pREE, predicted REE; FM, fat mass; FFM, fat-free mass.
(DOCX)
