# Supplementary material for: The relationship between resting energy expenditure and thyroid hormones in response to short-term weight loss in severe obesity
Source: PLoS One. 2018 Oct 19;13(10):e0205293. doi: 10.1371/journal.pone.0205293 (PMC6195261; doi:10.1371/journal.pone.0205293)
Supplement: S1 Table — legend. Significance between the two time points was obtained by paired T test and is depicted as: a, p<0.05; b, p<0.01; c, p<0.001. For abbreviations: BMI, body mass index; REE, resting energy expenditure; pREE, predicted REE; FM, fat mass; FFM, fat-free mass. (DOCX) [file pone.0205293.s001.docx]

**S1 Table: Baseline data in the obese population obtained at baseline and at the end of the 4-week study, and expressed as percent variation over baseline values.**

| **Variables** | **At study**  **entry (n=100)** | **At study**  **end (n=100)** | **Percent variation** |
| --- | --- | --- | --- |
| Age (yrs) | 40.4±12.7 | - | - |
| BMI (kg/m^2^) | 45.1±4.8^c^ | 42.5±4.4 | -5.5±1.8 |
| Waist (cm) | 128±12.2^c^ | 122.6±11.6 | -4.3±1.2 |
| TSH (mIU/L) | 2.09±0.92^c^ | 1.87±0.91 | -6.3±32.2 |
| FT3 (ng/L) | 3.22±0.36^c^ | 3.10±0.33 | -3.3±9.7 |
| FT4 (ng/L) | 11.7±1.59^c^ | 12.0±1.52 | 3.9±12.7 |
| FT3/FT4 ratio | 0.28±0.06^b^ | 0.26±0.03 | -5.7±13.4 |
| FM (%) | 46.7±6.8^a^ | 45.8±6.9 | -1.6±6.7 |
| FFM (kg) | 67.2±13^c^ | 64.5±12.5 | -3.7±7.7 |
| REE (Kcal/day) | 2038±372^c^ | 1936±322 | -4.1±10.8 |
| REE/pREE (%) | 94.1±11.0^a^ | 91.8±10.2 | -0.12±0.5 |
| REE/FFM (kcal/kg/day) | 30.7±4.7 | 30.4±3.9 | 0±12.3 |

Significance between the two time points was obtained by paired T test and is depicted as: a, p<0.05; b, p<0.01; c, p<0.001. For abbreviations: BMI, body mass index; REE, resting energy expenditure; pREE, predicted REE; FM, fat mass; FFM, fat-free mass.
