# Supplementary material for: The relationship between resting energy expenditure and thyroid hormones in response to short-term weight loss in severe obesity
Source: PLoS One. 2018 Oct 19;13(10):e0205293. doi: 10.1371/journal.pone.0205293 (PMC6195261; doi:10.1371/journal.pone.0205293)
Supplement: S2 Table — legend: Significance between the two subgroups was obtained by ANOVA. For abbreviations: BMI, body mass index; REE, resting energy expenditure; pREE, predicted REE; FM, fat mass; FFM, fat-free mass. (DOCX) [file pone.0205293.s002.docx]

**S2 Table: Data summary in the obese population at study entry stratified according to REE measured as lower vs higher than predicted REE.**

| **Variables** | **Low baseline REE**  **(N=69)** | **High baseline REE**  **(N=31)** | **p** |
| --- | --- | --- | --- |
| Age (yrs) | 40.6±12.7 | 40.0±12.8 | 0.8 |
| BMI (kg/m^2^) | 44.8±4.3 | 45.5±5.8 | 0.5 |
| Waist (cm) | 128±11.8 | 128±13.3 | 0.9 |
| TSH (mIU/L) | 2.09±0.93 | 2.09±0.9 | 0.9 |
| FT3 (ng/L) | 3.22±0.35 | 3.23±0.37 | 0.8 |
| FT4 (ng/L) | 11.8±1.72 | 11.2±1.09 | 0.08 |
| FT3/FT4 ratio | 0.27±0.05 | 0.28±0.07 | 0.8 |
| FM (%) | 46.2±6.8 | 48.0±6.7 | 0.2 |
| FFM (kg) | 67.9±13.1 | 65.2±12.7 | 0.3 |
| REE (Kcal/day) | 1951±338 | 2232±397 | 0.0001 |
| REE/pREE (%) | 88.9±7.5 | 107.4±10.2 | 0.0001 |
| REE/FFM (kcal/kg/day) | 29.1±4.3 | 34.5±3.5 | 0.0001 |

Significance between the two subgroups was obtained by ANOVA. For abbreviations: BMI, body mass index; REE, resting energy expenditure; pREE, predicted REE; FM, fat mass; FFM, fat-free mass.
