# Supplementary material for: The relationship between resting energy expenditure and thyroid hormones in response to short-term weight loss in severe obesity
Source: PLoS One. 2018 Oct 19;13(10):e0205293. doi: 10.1371/journal.pone.0205293 (PMC6195261; doi:10.1371/journal.pone.0205293)
Supplement: S3 Table — legend: Significance between genders at baseline and at the study-end time-points was assessed by ANOVA and is depicted as: a, p<0.05; b, p<0.01; c, p<0.001. Significance within genders between baseline and study-end time-points was assessed by paired T test and is depicted as: d, p<0.05; e, p<0.01; f, p<0.001. For abbreviations: BMI, body mass index; REE, resting energy expenditure; pREE, predicted REE; FM, fat mass; FFM, fat-free mass. (DOCX) [file pone.0205293.s003.docx]

**S3 Table: Data summary obtained at baseline and at the end of the 4-week study in the obese population subgrouped by gender.**

| **Variables** | **Men (n=50)** | | | **Women (n=50)** | | |
| --- | --- | --- | --- | --- | --- | --- |
|  | **At study**  **entry** | **At study**  **end** | **Percent variation** | **At study**  **entry** | **At study**  **end** | **Percent variation** |
| Age (yrs) | 43.1±12.2^a^ | - | - | 38±12.8 | - | - |
| BMI (kg/m^2^) | 44.7±4.2^f^ | 41.9±4 | -6.2±1.9^c^ | 45.3±5.1^f^ | 43.1±4.8 | -4.9±1.5 |
| Waist (cm) | 133.6±9.2^cf^ | 128±8.5^c^ | -4.1±2.5 | 123.5±12.6^f^ | 117.9±11.9 | -4.5±1.9 |
| TSH (mIU/L) | 2.12±0.36^e^ | 1.82±0.94 | -10.5±29.9 | 2.06±0.89 | 1.91±0.89 | -2.6±39.2 |
| FT3 (ng/L) | 3.33±0.36^b^ | 3.26±0.28^c^ | -1.6±9.1 | 3.12±0.33^e^ | 2.96±0.31 | -4.8±10.2 |
| FT4 (ng/L) | 11.96±1.92^f^ | 12.76±1.41^c^ | 8.3±14.8^b^ | 11.38±1.17 | 11.37±1.30 | 0.2±9.0 |
| FT3/FT4 ratio | 0.28±0.05^e^ | 0.26±0.03 | -7.6±13.9 | 0.27±0.05 | 0.26±0.04 | -3.9±12.8 |
| FM (%) | 41.5±6.2^ce^ | 39.7±4.3^c^ | -3.6±8.7^b^ | 51.3±3.1 | 51.4±3.1 | 0.1±3.3 |
| FFM (kg) | 77.9±9.7^ce^ | 75.5±8^c^ | -2.4±10.5 | 57.5±6.5^f^ | 54.6±5.8 | -4.9±3.5 |
| REE (Kcal/day) | 2247±337^ce^ | 2122±307^c^ | -4.7±12.3 | 1841±298^e^ | 1768±233 | -3.6±9.4 |
| REE/pREE (%) | 91.2±10.0^a^ | 88.2±10.9^b^ | -0.12±0.5 | 96.7±11.3 | 95±8.5 | -0.11±0.5 |
| REE/FFM (kcal/kg/day) | 29.1±4.8^c^ | 28.2±3.6 | -1.7±13.8 | 32.4±4.1 | 32.4±3.2 | 1.5±10.8 |

Significance between genders at baseline and at the study-end time-points was assessed by ANOVA and is depicted as: a, p<0.05; b, p<0.01; c, p<0.001. Significance within genders between baseline and study-end time-points was assessed by paired T test and is depicted as: d, p<0.05; e, p<0.01; f, p<0.001. For abbreviations: BMI, body mass index; REE, resting energy expenditure; pREE, predicted REE; FM, fat mass; FFM, fat-free mass.
