# Supplementary material for: The relationship between resting energy expenditure and thyroid hormones in response to short-term weight loss in severe obesity
Source: PLoS One. 2018 Oct 19;13(10):e0205293. doi: 10.1371/journal.pone.0205293 (PMC6195261; doi:10.1371/journal.pone.0205293)
Supplement: S4 Table — legend: For abbreviations: REE, resting energy expenditure. (DOCX) [file pone.0205293.s004.docx]

**S4 Table: Bivariate correlation analysis between thyroid function parameters and REE at baseline and at the study end.**

| **Variables** | **REE (kcal/day)** | | | |
| --- | --- | --- | --- | --- |
|  | **Baseline** | | **At study end** | |
|  | **r** | **p** | **r** | **p** |
| TSH (mIU/L) | 0.039 | 0.70 | -0.023 | 0.8 |
| FT3 (ng/L) | 0.199 | <0.05 | 0.240 | 0.02 |
| FT4 (ng/L) | 0.128 | 0.2 | 0.425 | <0.0001 |
| FT3/FT4 | -0.090 | 0.9 | -0.180 | 0.07 |

For abbreviations: REE, resting energy expenditure.
