# Supplementary material for: The relationship between resting energy expenditure and thyroid hormones in response to short-term weight loss in severe obesity
Source: PLoS One. 2018 Oct 19;13(10):e0205293. doi: 10.1371/journal.pone.0205293 (PMC6195261; doi:10.1371/journal.pone.0205293)
Supplement: S5 Table — legend: Between-subgroup significance at each time point was assessed by ANOVA and expressed as: a, p<0.05; b, p<0.01; c, p<0.001. Within-subgroup significance between the two time points was assessed by paired T test and expressed as: d, p<0.05; e, p<0.01; f, p<0.001. For abbreviations: BMI, body mass index; REE, resting energy expenditure; pREE, predicted REE; FM, fat mass; FFM, fat-free mass. (DOCX) [file pone.0205293.s005.docx]

**S5 Table: Data summary in the obese population stratified according to percent REE variation below (decreased REE) or above (increased REE) baseline values recorded at the end of the study.**

| **Variables** | **At study entry** | | **At study end** | |
| --- | --- | --- | --- | --- |
|  | **Decreased**  **REE** | **Increased**  **REE** | **Decreased**  **REE** | **Increased**  **REE** |
| No. | 66 | 34 | 66 | 34 |
| Gender (M/F) | 32/34 | 18/16 | 32/34 | 18/16 |
| Age (yrs) | 38.7±13.4^a^ | 44.0±10.5 | - | - |
| BMI (kg/m^2^) | 45.1±4.7^f^ | 44.9±4.8^f^ | 42.5±4.5 | 42.6±4.4 |
| Waist (cm) | 125.7±19.6^f^ | 126.4±12.4^f^ | 118.5±18.2 | 119.9±12.8 |
| TSH (mIU/L) | 2.16±0.96^d^ | 1.93±0.82 | 1.94±0.98 | 1.73±0.75 |
| FT3 (ng/L) | 3.23±0.37^e^ | 3.21±0.34 | 3.10±0.35 | 3.11±0.32 |
| FT4 (ng/L) | 11.58±1.64^d^ | 11.83±1.51 | 11.94±1.36 | 12.2±1.81 |
| FT3/FT4 ratio | 0.28±0.06^f^ | 0.27±0.06 | 0.26±0.04 | 0.26±0.03 |
| FM (%) | 47.3±7.3^d^ | 45.6±5.6 | 46.3±7.1 | 44.9±6.6 |
| FFM (kg) | 66.2±13.6^f^ | 69.0±11.8^f^ | 63.6±12.7 | 66.6±12.1 |
| REE (Kcal/day) | 2093.7±383.6^af^ | 1903.8±328.6^f^ | 1874.3±312.3^b^ | 2068.3±309.3 |
| REE/pREE (%) | 97.2±9.5^cf^ | 87.8±11.5f | 89.1±9.2^c^ | 97.5±10.2 |
| REE/FFM (kcal/kg/day) | 32.0±4.5^cf^ | 27.8±3.9^f^ | 29.8±3.7 | 31.5±4.3 |

Between-subgroup significance at each time point was assessed by ANOVA and expressed as: a, p<0.05; b, p<0.01; c, p<0.001. Within-subgroup significance between the two time points was assessed by paired T test and expressed as: d, p<0.05; e, p<0.01; f, p<0.001. For abbreviations: BMI, body mass index; REE, resting energy expenditure; pREE, predicted REE; FM, fat mass; FFM, fat-free mass.
